# Commentary: Association between albumin-to-alkaline phosphatase ratio and a 3-month unfavorable outcome in patients with acute ischemic stroke

**DOI:** 10.3389/fnut.2025.1678441

**Published:** 2025-11-19

**Authors:** Xiangqi Kong, Wei Jing

**Affiliations:** 1 Third Hospital of Shanxi Medical University, Taiyuan, China; 2 Shanxi Bethune Hospital, Shanxi Academy of Medical Sciences, Taiyuan, China; 3 Tongji Shanxi Hospital, Taiyuan, China

**Keywords:** acute ischemic stroke, albumin-to-alkaline phosphatase ratio, prognostic biomarker, functional outcome, commentary

## 1 Introduction

We read with great interest the article by Zhang et al., published in Frontiers in Nutrition, titled “Association between albumin-to-alkaline phosphatase ratio and a 3-month unfavorable outcome in patients with acute ischemic stroke” (1). The study investigates a novel and clinically relevant biomarker—the albumin-to-alkaline phosphatase ratio (AAPR)—and its association with short-term functional prognosis in patients with acute ischemic stroke (AIS). The large-scale cohort design and comprehensive statistical modeling are commendable. We would like to offer several constructive comments and suggestions that might help further strengthen the interpretation and clinical applicability of this valuable study.

### 1.1 Study population and generalizability

First, although the study included a large number of patients and used a well-structured design, the data were drawn from a single center in South Korea. The relatively homogeneous ethnic background may limit the generalizability of the findings to broader or more diverse populations. Further external validation in multi-ethnic, multicenter cohorts would be beneficial to establish broader clinical applicability.

### 1.2 Inconsistency in statistical interpretation

Second, in Table 3, the *p*-value for trend across AAPR tertiles is reported as < 0.001, which statistically indicates a significant linear trend. However, the authors state in the results section that “there were no statistically significant differences in the trends,” which appears inconsistent with the data. We suggest clarifying this statement to avoid misinterpretation and ensure alignment with the statistical results presented.

### 1.3 Threshold validity and clinical utility

Third, the reported inflection point (AAPR = 0.588) is an important feature of the study's non-linear findings. However, its clinical validity and robustness require further evaluation. We suggest conducting sensitivity analyses to assess whether this threshold holds across different subgroups or under alternative imputation strategies. Additionally, translating this threshold into a practical clinical tool—for example, by incorporating it into existing stroke risk stratification scores—could enhance the usability of AAPR in routine stroke management.

### 1.4 Neurological severity stratification

Fourth, the use of NIHSS < 6 vs. ≥6 as a binary cutoff may oversimplify the spectrum of neurological severity in AIS. The NIHSS is a well-established continuous measure, and more nuanced stratification may reveal additional associations between AAPR and clinical outcomes. Notably, the Centers for Medicare & Medicaid Services (CMS) and several large-scale studies have categorized NIHSS scores into five clinically meaningful groups (2): 0 (no measurable stroke symptoms), 1–4 (minor stroke), 5–15 (moderate stroke), 16–20 (moderate to severe stroke), and 21–42 (severe stroke). Applying such stratification could improve the clinical interpretability of subgroup findings and better reflect the heterogeneity of stroke severity.

### 1.5 Treatment-related confounding factors

Fifth, as a secondary data analysis, the study lacks information on critical treatment-related variables such as intravenous thrombolysis, mechanical thrombectomy, and rehabilitation intensity. These factors are known to influence post-stroke outcomes and could confound the observed association between AAPR and prognosis. Inclusion of such variables in future research would help better isolate the prognostic value of AAPR and reduce residual confounding.

### 1.6 Metabolic and hepatic influences

Sixth, several important metabolic parameters associated with serum ALP levels were not evaluated in the current study. Specifically, data on serum phosphate levels, 25-hydroxyvitamin D concentrations, and comorbid bone disorders were unavailable. These variables are closely linked to calcium and bone metabolism and may interact with ALP levels in ways that influence stroke outcomes (3). Moreover, the study did not include detailed information on hepatic diseases. Given that serum ALP consists of isoenzymes originating from bone, liver, intestine, and other tissues, the source-specific elevation of ALP remains unclear. Disentangling these isoenzymes through more precise assays in future studies could provide critical mechanistic insights into the observed associations and improve the specificity of AAPR as a prognostic biomarker.

### 1.7 Sensitivity analysis using E-values

Seventh, given the observational nature of this study and the potential for residual confounding—particularly from unmeasured treatment- or metabolism-related factors—we recommend the use of E-values to evaluate the robustness of the observed associations. The E-value quantifies the minimum strength of association that an unmeasured confounder would need to have with both the exposure and the outcome to fully explain away the observed relationship (4). Its application would provide additional insight into the sensitivity of the results and further strengthen the conclusions.

## 2 Discussion

Zhang et al. have presented a valuable and timely study highlighting the prognostic relevance of AAPR in acute ischemic stroke. Our suggestions—clarifying statistical interpretations, refining subgroup stratification, incorporating sensitivity analyses such as E-values, and exploring metabolic and isoenzymatic pathways—could substantially enhance the scientific rigor, reproducibility, and clinical translatability of the research. These measures are likely to improve the credibility and long-term utility of the study's conclusions in stroke risk stratification and management.
